# Characterisation of the genomic landscape of *CRLF2*‐rearranged acute lymphoblastic leukemia

**DOI:** 10.1002/gcc.22439

**Published:** 2017-01-18

**Authors:** Lisa J. Russell, Lisa Jones, Amir Enshaei, Stefano Tonin, Sarra L. Ryan, Jeyanthy Eswaran, Sirintra Nakjang, Elli Papaemmanuil, Jose M. C. Tubio, Adele K. Fielding, Ajay Vora, Peter J. Campbell, Anthony V. Moorman, Christine J. Harrison

**Affiliations:** ^1^ Leukaemia Research Cytogenetics Group, Northern Institute for Cancer Research, Newcastle UniversityNewcastle‐upon‐TyneUK; ^2^ Bioinformatics Support Unit, Newcastle UniversityNewcastle‐upon‐TyneUK; ^3^ Department of Epidemiology‐BiostatisticsMemorial Sloan Kettering Cancer CenterUSA; ^4^ Cancer Genome ProjectWellcome Trust Sanger InstituteHinxtonUK; ^5^ Research Department of HaemaolotyUCL Cancer InstituteLondonUK; ^6^ Department of HaematologySheffield Children's HospitalSheffieldUK

## Abstract

Deregulated expression of the type I cytokine receptor, *CRLF2*, is observed in 5–15% of precursor B‐cell acute lymphoblastic leukaemia (B‐ALL). We aimed to determine the clinical and genetic landscape of those with *IGH‐CRLF2* or *P2RY8‐CRLF2* (*CRLF2*‐r) using multiple genomic approaches. Clinical and demographic features of *CRLF2*‐r patients were characteristic of B‐ALL. Patients with *IGH‐CRLF2* were older (14 y vs. 4 y, *P* < .001), while the incidence of *CRLF2*‐r among Down syndrome patients was high (50/161, 31%). *CRLF2*‐r co‐occurred with primary chromosomal rearrangements but the majority (111/161, 69%) had B‐other ALL. Copy number alteration (CNA) profiles were similar to B‐other ALL, although *CRLF2*‐r patients harbored higher frequencies of *IKZF1* (60/138, 43% vs. 77/1351, 24%) and *BTG1* deletions (20/138, 15% vs. 3/1351, 1%). There were significant differences in CNA profiles between *IGH‐CRLF2* and *P2RY8‐CRLF2* patients: *IKZF1* (25/35, 71% vs. 36/108, 33%, *P* < .001), *BTG1* (11/35, 31% vs. 10/108, 9%, *P* =.004), and *ADD3* deletions (9/19, 47% vs. 5/38, 13%, *P* =.008). A novel gene fusion, *USP9X‐DDX3X*, was discovered in 10/54 (19%) of patients. Pathway analysis of the mutational profile revealed novel involvement for focal adhesion. Although the functional relevance of many of these abnormalities are unknown, they likely activate additional pathways, which may represent novel therapeutic targets.

## 1 Introduction

Acute lymphoblastic leukaemia (ALL) is defined by primary chromosomal abnormalities that drive disease progression, with impact on prognosis and treatment stratification.^1^ One quarter of patients, known as B‐other ALL, lack a known primary abnormality.^1^ A group of B‐other patients, known as Ph‐like*/BCR‐ABL1*‐like, constitute 10–15% of B‐ALL. Although they lack the *BCR‐ABL1* fusion, their gene expression profile is similar to *BCR‐ABL1* positive ALL.^2^, ^3^ They are characterised by high expression of the type I cytokine receptor, cytokine receptor‐like factor 2 (*CRLF2*), the presence of tyrosine kinase fusion genes and mutations of genes within the *JAK/STAT* and *RAS* signaling pathways.^4^ Deregulated expression of *CRLF2* (*CRLF2*‐d) is observed in 27–50% of patients with *BCR‐ABL1*‐like disease (5–15% of B‐ALL).^1^, ^4^, ^5^, ^6^ Deregulation occurs via three genomic rearrangements (*CRLF2*‐r): a cryptic reciprocal translocation with the immunoglobulin heavy chain locus (*IGH*); an interstitial deletion within the pseudoautosomal region (PAR1) of chromosomes X and Y (*P2RY8‐CRLF2*); rare but recurrent *CRLF2* mutations. All three *CRLF2*‐r result in overexpression of *CRLF2* mRNA and protein; however, alone they are insufficient to cause overt leukaemia.^5^, ^7^ Interestingly, studies identifying *CRLF2*‐d patients by mRNA and protein expression have shown that some patients do not harbor one of the three known genomic rearrangements.^8^ The cause of this overexpression is currently unknown. The incidence of *CRLF2*‐r is high in patients with Down syndrome ALL (DS‐ALL) (>50%) and intrachromosomal amplification of chromosome 21 (iAMP21) (25%),^5^, ^7^, ^9^ ^−11^ with other established cytogenetic abnormalities rarely associated. However, data indicating whether *CRLF2*‐r is a primary or secondary event are scarce.^12^ It is well documented that deletions of genes involved in B‐cell differentiation and cell cycle control are recurrent in these patients.^5^, ^13^, ^14^, ^15^ Mutations of the Janus kinase family, in particular *JAK2*, and mutations of *IL7R*, are also recurrently observed and together result in IL‐3 independent growth of mouse BaF3 cells.^11^, ^16^ Recently, mutations affecting other kinase genes have also been reported in *CRLF2*‐d ALL.^4^, ^17^ Both *BCR‐ABL1*‐like and *CRLF2*‐d ALL have been associated with poor outcome and increased risk of relapse.^2^, ^3^, ^10^ Although MRD‐directed treatment intensification improves survival,^18^ outcome for *CRLF2*‐d ALL remains less favorable than for patients with good risk cytogenetics. Hence, novel therapeutic strategies are required to improve survival and quality of life. In this study, we have explored the clinical and genetic landscape of patients with known *CRLF2* rearrangements. The aims of this study were: (1) to identify the clinical and genomic differences that may exist between patients with *IGH* or *P2RY8* driven overexpression of *CRLF2; (*2) to undertake pathway analysis of whole genome (WGS) and whole exome sequencing (WES) data to highlight additional pathways that may co‐operate with rearrangements of *CRLF2*.

## 2 Materials and methods

### 2.1 Patient samples

We identified 172 patients with *CRLF2‐*r ALL by fluorescence in situ hybridisation (FISH) and multiplex ligation‐dependent probe amplification (MLPA) from the following trials: UKALL97/99 (*n* = 68), UKALL2003 (*n* = 75), UKALLXI (*n* = 6), and UKALLXII (*n* = 26) (Supporting Information Table 1). The patients included in this study were identified by screening for *CRLF2*‐r^6^, ^14^, ^19^, ^20^, ^21^ and were representative of the trial. Demographic and clinical details were collected by the Clinical Trial Service Unit (CTSU, Oxford University, UK). Each contributing centre obtained relevant ethical approval. Informed consent was obtained in accordance with the Declaration of Helsinki. Diagnostic immunophenotypes were collected centrally with review of original reports for 50 patients (Supporting Information Table 2).

### 2.2 Cytogenetics and fluorescence in situ hybridisation

For details on genetic testing, see Supporting Information Figure 1. Karyotype data were collected from UK cytogenetics laboratories (Supporting Information Table 1). Cytogenetic analysis and FISH were carried out on the same diagnostic patient samples. The involvement of *IGH* was determined using the LSI IGH Dual Color Break‐Apart Rearrangement Probe (Abbott Molecular, Green Oaks, IL).^6^, ^22^ Additional FISH probes used in this study are shown in Supporting Information Table 3. Five control slides of fixed cells from normal individuals were hybridised with all probe combinations^5^ (Supporting Information Table 3) to determine the cut‐off percentages for false positive results (± 3 × standard deviations). A minimum of 100 nuclei were scored by eye for each FISH test by two independent analysts. When combining three or more florophores, capture and scoring was carried out using an automated Olympus BX‐61 8‐bay stage florescence microscope. Images were analysed using the CytoVision 7.1 SPOT counting system (Leica Microsystems, Gateshead, UK).

### 2.3 Multiplex ligation‐dependent probe amplification

Copy number alterations (CNA) were investigated (*n* = 154) by MLPA using the SALSA MLPA kit P335 (MRC Holland, Amsterdam, Netherlands) according to manufacturer's guidelines as previously reported.^15^

### 2.4 Affymetrix genome‐wide human SNP6.0 array

Sufficient material was available from 26 (representitive for cohort) *CRLF2*‐r patients (15 with matched germ‐line sample) for Affymetrix Genome‐Wide Human SNP6.0 analysis, performed by AROS Applied Biotechnology A/S (Aarhus, Denmark). Copy number alterations were analysed using Genotyping Console (Affymetrix software) with additional manual curation (GEO accession number GSE83272).

### 2.5 Low depth paired‐end and whole exome sequencing

Sufficient diagnostic DNA was available from 11 *CRLF2*‐r patients (representitive of cohort and all patients have SNP analysis completed), which was prepared for library construction (300–500 bp), flow cell preparation and cluster formation using the Illumina no‐PCR library protocol (Illumina Inc, San Diego, CA), and 50 bp reads were performed using the Illumina Genome Analyzer IIx instrument following the manufactures guidelines. Structural variants were selected as previously reported.^23^

Sufficient diagnostic and germ‐line DNA from the same 11 patients was prepared for Illumina paired‐end sequencing with subsequent exome enrichment using the Agilent SureSelect Human All Exon 50 MB kit (Agilent Technologies LTD, Berkshire, UK). Guidelines for the Illumina Genome Analyzer IIx instrument were followed for flow‐cell preparation, cluster generation and paired‐end sequencing of 75 bp reads.^23^, ^24^ Whole genome and exome sequencing data are available using EGA accession numbers EGAD00001002007 and EGAD00001002008, respectively.

### 2.6 Structural variant detection and validation

Sequencing reads were mapped to the human genome (hg19) with a minimum and mean fold coverage of 5.61 and 8.46, respectively. The algorithm BRASS^25^ was used to identify groups of ≥2 discordantly mapped (distance or orientation) paired‐end reads. Rearrangements observed in 5 or more paired‐end reads were validated (excluding those where both reads were in the same intron or outside the coding regions) by conventional PCR and Sanger sequencing using diagnostic and remission DNA. If a rearrangement failed to validate using two sets of primers, repeated twice over a temperature gradient, it was regarded as a fail. Breakpoints within immunoglobulin loci were not validated. MLPA and SNP data were also used to validate CNA. A total of 476 SV were identified from WGS of 11 patients with *CRLF2*‐r ALL (average 43 SV/patient, range 22–90), including 416 intrachromosomal and 57 interchromosomal rearrangements.

### 2.7 Mutation and in/del detection and validation

A total of 458 SNVs and insertion/deletions (in/dels) were identified from the same 11 patients. A minimum sequencing coverage of 30‐fold was required for each sample. Default setting of BWA^24^ and CaVEMan was used to align the reads and detect somatically acquired single nucleotide substitutions as previously reported^23^. The algorithm PINDEL^26^ was used to detect in/dels.^23^ Conventional PCR was used to validate the substitutions, with subsequent 454 pyrosequencing for confirmation.^23^ Conventional PCR validated the in/dels, with subsequent sequencing using the ABI terminator Cycle Sequencing Kit (Applied Biosystems) to confirm each in/del.^23^

### 2.8 Targeted JAK mutation screening

Primers were used to amplify exon 14 of the *JAK2* and *JAK1* genes only^27^ and Sanger sequencing confirmed the presence of the mutation. As far as we are aware *CRLF2* mutations have not been reported in patients with *IGH‐CRLF2* or *P2RY8‐CRLF2*, therefore screening for these mutations was not carried out.

### 2.9 Statistical analysis

The distribution of categorical variables was examined using Fisher's exact test. Due to the investigative nature of this analysis, we did not apply stringent multiple comparisons adjustment (all tests were conducted at the 5% significance level). All analyses were performed using Intercooled Stata 14.1 for Windows (Stata Corporation, College Station, TX).

### 2.10 Pathway analysis

Pathway mapping was performed on a defective gene list identified for each sample. A gene was considered to be defective if: (1) it contained at least one nonsilent mutation, (2) was located at the break point junction of the identified structural variants, and (3) was located within a defective region (detected either from SNP6 or WGS) of no more than 1 MB in size. Gene lists from the defective regions were retrieved from Human reference genome hg19 via biomart^28^ and defective gene lists were mapped to KEGG pathways^29^ using R/Bioconductor package KEGGREST (Tenenbaum D. KEGGREST: Client‐side REST access to KEGG. R package version 1.11.0). Pathway enrichment analysis was determined using hypergeometric test. The test is based on the probability of observing *x* number of genes from a given pathway as being defective, given a process of sampling without replacement of all protein‐coding genes (20805) annotated in the human genome (GRCh37.p13, INSDC Assembly GCA_000001405.14, Feb 2009). This gene list is considered as a representative gene set that are mutated among *CRLF2*‐r cases. The basic assumption for the analysis is that mutations among *CRLF2*‐r cases arise randomly, so the enrichment test is used as a statistical method to test whether there is any selective pressure that makes mutations occur in particular pathways more often than expect by chance. Enrichment tests were performed with the defective gene lists from all samples as well as for each subtype separately.

## 3 Results

### 3.1 The clinical and demographic features of *CRLF2*‐r patients are characteristic of B‐ALL

**Figure 1:**
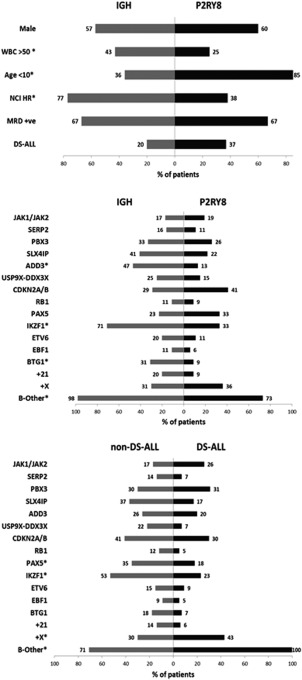
Comparative histograms of recurrent genetic abnormalities in patients with *CRLF2*‐r ALL. (A) Comparison of clinical data between patients with *IGH‐CRLF2* (gray bars) and *P2RY8‐CRLF2* (black bars). (B) Comparison of genetic data between patients with *IGH‐CRLF2* (gray bars) and *P2RY8‐CRLF2* (black bars). (C) Comparison between *CRLF2*‐r DS‐ALL (black bars) and *CRLF2*‐r non DS‐ALL patients (gray bars). * denotes those abnormalities where the incidence between the two groups is statistically significant

All patients with *CRLF2*‐r were B‐ALL (*n* = 172): *IGH‐CRLF2* (*n* = 47) and *P2RY8‐CRLF2* (*n* = 125), median age 5 years (range 1–60 y), male predominance (59%), 70% had WBC <50 × 10^6^/l (Supporting Information Table 4; Figure 1A). The age distribution was similar to B‐ALL overall, with the majority <10 years (71%).^20^ Median age (4 y vs. 14 y, *P* < .001), WBC (25% vs. 43% with WBC >50 × 10^6^/l, *P* = .016) and percentage NCI high risk (38% vs. 77%, *P* < .001) differed significantly between *P2RY8‐CRLF2* and *IGH‐CRLF2,* respectively (Supporting Information Table 4; Figure 1A). We confirmed the high frequency of DS‐ALL among *CRLF2*‐r ALL (31%, 50/161) (DS status not available for 11 patients), with more DS‐ALL patients harboring the *P2RY8‐CRLF2* fusion (*P2RY8‐CRLF2*, *n* = 41 vs. *IGH‐CRLF2 n* = 9, 35% vs. 20%, respectively, *P* = .087) (Supporting Information Table 4; Figure 1B).

Cytogenetic analysis was successful in 160 *CRLF2*‐r patients. Normal karyotype was observed in 51 (36%) patients. The majority, including those with DS‐ALL, were classified as B‐other ALL (Figure 1B,C); however, primary, established chromosomal abnormalities co‐occurred in 22% (Supporting Information Table 1): *ETV6‐RUNX1* (*n* = 3), *BCR‐ABL1* (*n* = 5), high hyperdiploidy (*n* = 12), and iAMP21 (*n* = 17). All but one of these patients had *P2RY8‐CRLF2* (*n* = 36) (Figure 1B). No patients had t(1;19)(q23;p13), *KMT2A* (*MLL*) rearrangements or *ABL*‐class fusions involving *ABL1*, *ABL2*, *PDGFRB,* and *CSF1R*.^4^, ^30^, ^31^

### 3.2 Additional chromosomal abnormalities in *CRLF2*‐r patients

Recurrent somatic structural and numerical aberrations were present in 109 patients (Supporting Information Figure 2), including gains of chromosomes X (37/83, 45%), 21 (13/83, 16%), 17 (7/83, 8%), and 9 (4/83, 5%), in patients with and without DS. The gain of chromosome X was significantly enriched in *CRLF2‐*r patients, particularly among DS‐ALL, when compared to a cohort of B‐other patients, where only 5 of 1019 (5%) patients had gain of X (Supporting Information Table 5; Figure 1C).

Copy number alterations (CNA) were detected among patients with *CRLF2*‐r ALL by MLPA (*n* = 154) and SNP6.0 arrays (*n* = 26) (Table 1; Supporting Information Table 1 and 6‐7; Figure 1B). Deletions of *IKZF1*, *CDKN2A/B, PAX5,* and *BTG1* were present in 43% (*n* = 60), 38% (*n* = 52), 30% (*n* = 41), and 14% (*n* = 20) of cases, respectively. Deletions of *IKZF1* and *BTG1* occurred at higher incidences than seen in B‐other ALL at 23% and 2%, respectively.^21^ Deletions of *IKZF1* (71% vs. 33% *P* < .001) and *BTG1* (31% vs. 9%, *P* = .004) were more frequent in *IGH‐CRLF2* than *P2RY8‐CRLF2* patients, respectively (Figure 1B). There was a lower incidence of *PAX5* and *IKZF1* deletions in patients with DS‐associated *CRLF2*‐r ALL compared to those without DS (18% vs. 35% *P* < .047 and 23% vs. 53%, *P* = .003, respectively) (Figure 1C, Supporting Information Table 5). In fact, *CRLF2*‐r DS patients were more likely to have none of these deletions (48% vs. 15%, *P* < .001), with 89% (39/44) having fewer than three gene deletions.

**Table 1:** Focal aberrations identified by SNP, paired‐end and exome sequencing

**Patient ID**	**B‐cell differentiation**	**Cell cycle**	**Kinase**	**RAS**	**Cell adhesion**	**TP53**	**Other interesting genes**
***P2RY8‐CRLF2***							
9534		*BTG1, CDKN2AB*	*P2RY8‐CRLF2, JAK2, IL7R*	*NRAS*	*LAMA1*	*CDKN2AB*	*NFATC4, NCOA3*
11538	*ETV6, IKZF1, PAX5*	*CDKN2AB, FOXN3*	*P2RY8‐CRLF2, MAST4, STK38L, RPS6KA5*	*NRAS*	*ITGBL1, USH2A*	*CDKN2AB*	*CRIPAK, KDM4A, SKIL, CCDC88C, SMEK1, FBLN5, CYP4A11*
11706	*ETV6*		*P2RY8‐CRLF2, CKMT1A, SPHKAP, NBEA, PTPRT*		*PTPRT*		*CREBBP, GLI1*
20638		*CDKN2AB*	*P2RY8‐CRLF2, ACVRL1, IL7R*		*ITGA7, ANK2*	*CDKN2AB*	*MLLT3, TUSC1, TOPORS, DDX58, APTX*
20753	*IKZF1, PAX5*	*CDKN2A*	*P2RY8‐CRLF2, MAPK10*		*USH2A*	*CDKN2A*	*PPP2R3B, ZEDB1, IL9R*
21819	*IKZF1, PAX5, VPREB1*	*CDKN2AB, MCC*	*P2RY8‐CRLF2, INSRR*	*NF1*		*CDKN2AB*	*DDX6, CASZ1*
21991	*ETV6, VPREB1*	*CDKN2AB, CCND3*	*P2RY8‐CRLF2*		*CD38, NPNT*	*CDKN2AB*	*ARID5A, SEMA4C, BTLA, TBL1XR1, FHIT, PAXIP1*
***IGH‐CRLF2***							
11543	*EBF1, IKZF1*	*BTG1*	*IGH‐CRLF2, JAK2, TTBK1*				*NOX4, CHORDC1*
19599	*IKZF1, VPREB1*	*CDK13*	*IGH‐CRLF2, JAK2, CDK13*		*DST*		*TOP3A, CREB5, TRL4, CHN2, MPLKIP, INHBA, GLI3, TSC22D1*
21245	*EBF1, ETV6, IKZF1, PAX5, VPREB1*	*RB1, CDK6*	*IGH‐CRLF2, JAK2, ERBB4*		*CTNNA2*	*RB1*	*ABRA, BTLA, AFF1, MEF2C, ADD3, RUNX1*
21470	*ETV6, IKZF1, VPREB1*		*IGH‐CRLF2, CRLF2, PTPN11, RHBDL2*	*PTPN11*	*ITGB7, LAMC2, COL3A1, CTTNBP2*		*MIR181B1, MIR181A1, CADM1*

Other recurrent CNA observed from SNP6.0 arrays included: the histone cluster at 6p22.2 (*n* = 7), *VPREB1* (*n* = 6), *ADD3* (*n* = 5), *BTLA* (*n* = 4), *SLX4IP* (*n* = 3), *SERP2* and *TSC22D1* (*n* = 3), and *PBX3* (*n* = 2). Publically available SNP6.0 data^32^ showed these deletions to be present in other ALL subtypes: *ADD3*, *n* = 9 (4.69%), *SLX4IP*, *n* = 9 (4.69%), *BTLA*, *n* = 13 (6.77%). To verify their frequency in *CRLF2*‐r ALL, we screened additional samples by FISH, as fixed cells were the most abundant source of patient material, that also allowed detection of low level populations. However, the resolution of FISH restricted accurate detection of deletions <40 kb. Combined FISH and SNP6.0 data generated incidences of; *ADD3* (14/57, 25%), *SLX4IP* (13/44, 30%), *SERP2* and *TSC22D1* (7/55, 13%), and *PBX3* (16/56, 29%), with no difference in the prevalence between DS and non‐DS *CRLF2*‐r ALL. *IGH‐CRLF2* patients had a higher incidence of *ADD3* deletions (46% vs. 13%, *IGH* vs. *P2RY8, P* = .008) (Supporting Information Table 6; Figure 1B).

### 3.3 A novel fusion between *USP9X* and *DDX3X*

An interstitial deletion of chromosome sub‐band Xp11.4, fusing *USP9X* to *DDX3X* (Supporting Information Figure 3A), was observed in 2/26 cases by SNP6.0 arrays and 7/24 cases by FISH, including one DS patient, giving an incidence of 19% (Supporting Information Figure 3B). No cases were identified among the publically available SNP6.0 data.^32^ FISH revealed that the fusion was present in both minor and major clones (8‐91% of nuclei) in both male (*n* = 4) and female patients (*n* = 5). All male patients had either an additional copy of normal chromosome X or derived X involved in the *CRLF2*‐r. It was identified in patients with both *IGH* and *P2RY8* involvement (4 vs. 5, respectively). Real‐time PCR confirmed that the fusion was in‐frame and was expressed at the mRNA level (Supporting Information Figure 3C). Sanger sequencing verified fusion of *USP9X* exon 31 to exon 2 of *DDX3X* (Supporting Information Figure 3D). The DNA breakpoint sequence from three patients consistently fell within intron 31 of *USP9X* (covering a 3.5 kb region) and intron 1 of *DDX3X* (covering a 2.1 kb region).

### 3.4 Somatically acquired structural variants are rare, while the incidence of kinase and *JAK* mutations is high in *CRLF2*‐r ALL

**Figure 2:**
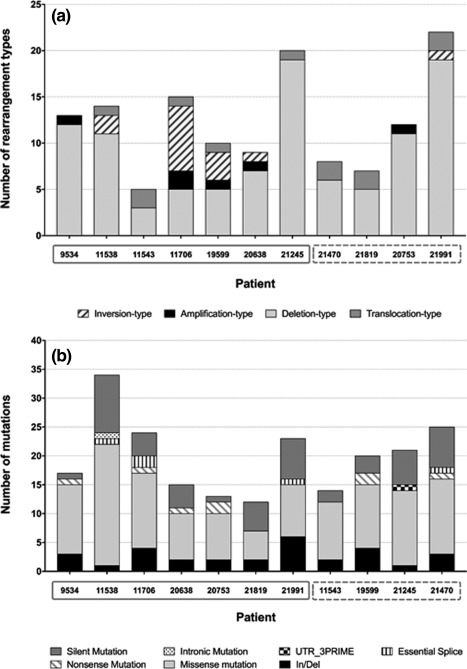
Acquired mutations and structural variations in *CRLF2*‐r ALL. (A) Histogram representing the distribution of structural variants (SV) in 11 patients with *CRLF2*‐r ALL (*x*‐axis, solid gray box depicts patients with *P2RY8‐CRLF2* and the dashed gray box, patients with *IGH‐CRLF2*), detected by paired‐end sequencing and validated by FISH, MLPA, SNP arrays and/or PCR and subsequent Sanger sequencing for breakpoint locations. Each patient is represented by a bar with the height of each bar being representative of the number of aberrations present in that patients sample. Different shades/patterns denote the different types of SV that were detected. (B) Histogram representing the distribution of coding mutations in same 11 patients with *CRLF2*‐r ALL (*x*‐axis, solid gray box depicts patients with *P2RY8‐CRLF2* and the dashed gray box, *IGH‐CRLF2* patients), detected by whole exome sequencing. Each patient is represented by a bar with the height of each bar being representative of the number of aberrations present in that patients sample. Different shades/patterns denote the different types of coding mutations that were detected

The somatic nature of 137 structural variants (SV) (6 tandem duplication‐, 104 deletion‐, 14 inversion‐, and 13 translocation‐type rearrangements) were validated, providing an average of 12.7 SV per patient, irrespective of DS status (Figure 2; Supporting Information Table 8; Supporting Information Figure 4). No novel recurrent rearrangements or fusion genes typical of B‐other ALL were identified in these 11 patients. There was no difference in the number of SV between patients with *IGH‐* and *P2RY8‐CRLF2* and also between patients with and without DS.

**Figure 3:**
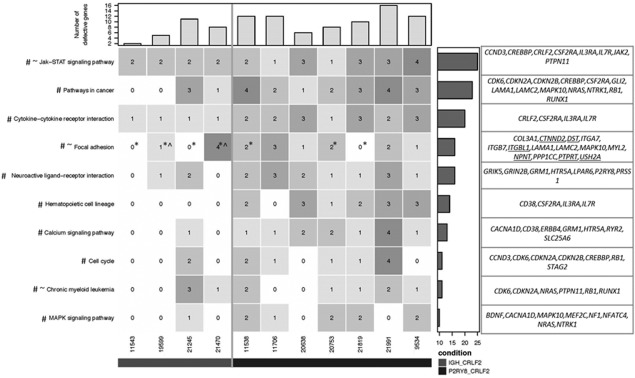
Heatmap showing the frequency distribution of defective genes. The top ten pathways in which defective genes are most frequently identified across 11 patients with *CRLF2*‐r ALL are shown. The number in each cell represents numbers of defective genes identified for particular pathways and patients, ranging from 0 (white) to 4 (dark gray). Total numbers of defective genes for each patient are summarized in the bar chart on the top panel of the heatmap. Note that genes which are members of two or more pathways were counted only once. Bar chart on the right panel summarises the total number of defective genes identified across the 11 patients for a given pathway (KEGG pathways, with additional manually annotated genes underlined). # significantly enriched (hypergeometric test *P*‐value < .05) in patients with *P2RY8‐CRLF2*; ∼ significantly enriched in patients with *IGH‐CRLF2*; * CNA of *IKZF1*; ^ *IKZF1* point mutation

WES from the same 11 patients detected 218 mutations, 187 point mutations, and 31 in/del events. Among the point mutations, 122 were predicted as possibly disease causing by PolyPhen‐2 and Mutation Taster (Figure 3; Supporting Information Table 9; Supporting Information Figure 4). The average number of mutations was 19.8 (point mutations alone: 17) per patient, with no difference between *IGH‐* and *P2RY8‐CRLF2* (*P* = .85) or those with or without DS (*P* = .41). In the majority of patients, at least half of the mutations were at variant allele frequency (VAF) higher than 30%; only one patient (#11706) had a higher number of mutations at a VAF of <20%. Recurrent mutations were identified in *CACNA1D*, *IKZF1*, *JAK2*, *IL7RA*, *NRAS,* and *USH2A* (*n* = 2 each). Ten patients had missense or nonsense point mutations in genes with protein kinase functions (Table 1), with six patients harboring either a mutation or CNA in two or more kinase genes.

Targeted screening for *JAK2* (*n* = 75) and *JAK1* (*n* = 36) mutations identified an incidence of 35% and 14%, respectively. There was no difference in the number of *JAK1* or *JAK2* mutations between *IGH‐* and *P2RY8‐CRLF2* (17% vs. 19%, *P* = .46) or DS and non‐DS patients (*JAK2* Ex14, 44% vs. 35%, *P* = .613; *JAK1* Ex14, 17% vs. 11%, *P* = 1; Supporting Information Table 5; Figure 1B,C). Due to selective screening of only exon 14 in the majority of patients, these numbers may represent an underestimate of the true incidence of *JAK1* and *JAK2* mutations in *CRLF2*‐r ALL.

### 3.5 Involvement of cell adhesion mutations in *CRLF2*‐r patients

WES data identified ≥1 somatic point mutation in a gene(s) involved in cell adhesion in eight patients including; *NPNT, ITGB7, COL3A1, ITGA7, ITGBL1, DST, LAMC2, PTPRT, MAPK10, CCND3, CTNNA2, CTNND2,* and *LAMA1* in single patients and *USH2A* in two patients (Table 1).

### 3.6 Pathway analysis reveals deregulation in known and novel pathways

Pathway analysis revealed those pathways likely to be deregulated due to the presence of mutations (not including silent mutations), in/dels and focal SV <1 Mb in size. The top ten pathways in which defective genes were most frequently identified are depicted in Figure 3. Unsurprisingly, *JAK‐STAT* involvement was seen in all patients. Other pathways included those involved in cancer, cytokine‐cytokine interaction, haematopoietic cell lineage and MAPK signaling. Interestingly, the focal adhesion pathway was also recurrently involved.

## 4 Discussion

In this study investigating the genomic landscape of *CRLF2*‐r ALL, we have confirmed the high incidence of *CRLF2*‐r in DS‐ALL, demonstrated its co‐existence with other primary chromosomal rearrangements and enrichment of specific chromosomal gains and deletions of *IKZF1*, *BTG1*, *ADD3, SERP2, TSC22D1, SLX4IP,* and *PBX3*. There were significant differences in CNA profiles between *P2RY8‐CRLF2* and *IGH‐CRLF2* patients with an increased incidence of *IKZF1*, *BTG1,* and *ADD3* deletions and a higher age at diagnosis being observed in the latter. These disparities correlate with reported differences in outcome, where for example older age and *IKZF1* status drive the inferior prognosis observed in adult *IGH‐CRLF2*.^14^ A fifth of *CRLF2*‐r patients harbor a primary chromosomal abnormality with all but one being *P2RY8‐CRLF2,* suggesting a co‐operating role for *CRLF2* deregulation. Interestingly, the sole patient with *IGH‐CRLF2* and a primary abnormality had the *BCR‐ABL1* fusion in a separate clone.^22^ Collectively, these data do not imply that *IGH* and *P2RY8* are driving distinct subgroups but rather that *CRLF2*‐d may play a dual role as both a primary and co‐operating driver in ALL, with the latter being more prominent among *P2RY8‐CRLF2* patients. This model is akin the *BCR‐ABL1* fusion, which although predominatly a primary driver, has also been reported as a secondary abnormality^22^, ^33^. Independent pathway analysis of the aberration profile of *IGH‐CRLF2* and *P2RY8‐CRLF2* patients found enrichment of all top ten pathways in the *P2RY8‐CRLF2* patients and only a subset of pathways in *IGH‐CRLF2* patients. Previous studies have reported the involvement of MAPK signaling in *CRLF2*‐r ALL.^34^ This pathway was not significantly enriched in our *IGH‐CRLF2* patients; however, this observation was based on only four patients and thus requires further validation. WES identified a frequency of 20 mutations per patient, similar to iAMP21‐ALL,^35^ but higher than other subgroups,^23^, ^36^, ^37^, ^38^. The high VAF of kinase mutations suggested that they were clonal and were likely acquired early in disease development. Mutations in *ERBB4*, *TTBK1,* and *STK38L* occurred within the catalytic domain with potential for constitutive activation. These mutations may activate *STAT5* (*ERBB4),* deregulate alternate pathways including, NFKB and chromosome alignment pathways (*TTBK1*), or regulate the Hippo signaling pathway (*STK38L*) by binding *MOB* kinase activators, which have tumour suppressor roles. Taken together, these data imply that *CRLF2*‐r may define a distinct subgroup of B‐other ALL.

For the first time, we have implicated a role for *USP9X* and *DDX3X* in *CRLF2*‐r ALL. This *USP9X‐DDX3X* fusion removes the ubiquitin carboxyl‐terminal hydrolase domain of *USP9X*, the promoter of *DDX3X*, multiple regulatory elements, MIR7641‐2 and non‐coding RNAs. The involvement of *USP9X* and *DDX3X* in cancer is known^39^, ^40^, ^41^, ^42^, ^43^, ^44^, ^45^ and another translocation involving *USP9X* has been reported in a *BCR‐ABL1*‐like ALL patient without *CRLF2*‐r.^4^ In keeping with the emerging functions of *USP9X*, its overexpression has been reported in B‐ALL, suggesting an oncogenic role.^46^ Knockdown of USP9X sensitises both prednisolone sensitive and resistant cell lines to glucocorticoid (GC)‐induced apoptosis, suggesting that reduced levels of USP9X may sensitise them to prednisolone treatment.^46^

With the success of targeted approaches using tyrosine kinase inhibitors (TKI) in *BCR‐ABL1‐*positive disease, there is clear rational to apply similar targeted approaches to other ALL subtypes. *CRLF2*‐r activate targetable pathways, including *JAK‐STAT*, *PI3K,* and *MAPK* signaling, and many groups are now assessing the efficacy of inhibitors to these pathways. The first inhibitor to be tested in *CRLF2*‐r ALL was the JAK1/2 inhibitor, Ruxolitinb.^47^ While this study showed a response in vivo, greater effect was observed in JAK‐activated, non‐*CRLF2*‐r ALL. Evidence of resistance to type I inhibitors in model systems of B‐ALL is already driving the development of type II JAK inhibitors.^48^ Targeting of mulitple pathways was an approach taken by Suryani et al, (2015) in assessing the JAK inhibitor, AZD1480, alone and in combination with the MEK inhibitor, Selumetinib.^49^ While this study showed a strong anti‐leukaemic effect in vitro, only modest effects were seen in vivo, an important consideration for future preclinical testing, which highlights the need to identify other common targets to provide further options for more patients. In addition to *JAK‐STAT*, we have identified several deregulated pathways in *CRLF2*‐r ALL, for example, focal adhesion signaling, one which has not previously been implicated in this subtype of ALL and was enriched in patients with both *IGH*‐ and *P2RY8‐CRLF2* ALL.

Mutations within genes controlling cell adhesion would seem to be more relevant in solid tumours, where such lesions would provide cells with a migratory and metastatic advantage. However, the presence of such mutations within the primary site may be advantageous, where small amounts of local movement can alter tumour growth and dispersal. In our study, eight patients (72%) had one or more focal adhesion gene mutations; an incidence much higher than reported in other ALL subtypes (*ETV6‐RUNX1*, 12%^23^, Ph‐like non‐*CRLF2*‐r, 15%^4^, low hypodiploidy, 5%^37^, high hyperdiploidy, 9.8%^38^, *KTM2A‐*rearranged infant ALL, 4.5%^36^). Mutated genes were specifically located within the laminin‐integrin‐MAPK cell signaling axis. The laminins (LAMA1 and LAMC2) and their receptor integrins (ITGA7, ITIBL1, and ITGB7) play a central role in cell proliferation and tumour cell invasiveness through their critical role in basement membrane adhesion.^50^ Although the functional consequence of these mutations in *CRLF2*‐r ALL remains to be determined, when laminin binding integrin signaling is altered, it activates downstream signaling kinases, such as FAK (focal adhesion kinase) and JUN (MAPK10)‐FOS, which impacts on cell proliferation and migration in various cancers.^51^, ^52^ In a recent study, the presence of the Ikaros isoform 6 (IK6), *IKZF1* haploinsuficiency or mutations in *BCR‐ABL1*‐positive disease have been shown to increase expression of adhesion molecules and increased transcription of *FAK*, rendering these cells insensitive to tyrosine kinase inhibition.^53^ The application of inhibitors to FAK in patients with *BCR‐ABL1*‐positive ALL resulted in the same abrogation of adhesion and self renewal programmes.^54^ Interestingly, all 11 patients with sequencing data either showed mutation/CNA of *IKZF1* or a mutation within a focal adhesion gene. Our data along with emerging literature in leukaemia highlight a role for the focal adhesion pathway in *CRLF2*‐r ALL.

In summary, we show clear clinical and genomic differences between patients with *IGH‐* and *P2RY8‐CRLF2*. We describe these rearrangements in the presence of other established cytogenetic abnormalities, suggesting a secondary role for *CRLF2*‐r in some patients, akin to *BCR‐ABL1* positive disease. However, due to low patient numbers, we were unable to ascertain any impact of *P2RY8‐CRLF2* on the prognosis of these patients. This co‐occurrence needs to be further assessed in a larger patient cohort. The treatment of patients across four independent trails precluded meaningful survival analysis between patients *IGH*‐ and *P2RY8*‐*CRLF2*. It is clear from our data that *CRLF2*‐r ALL is heterogeneous, requiring a combination of genetic abnormalities in functionally relevant genes to co‐operate with deregulated expression of *CRLF2*. Although the functional relevance of some of the deletions and mutations presented in this descriptive study are currently unknown, there are pointers to activation of additional targetable pathways. There is a clear requirement for studies addressing the biological effect of these aberrations, which together with the identification of mutations in cell adhesion genes and a high incidence of *IKZF1* deletions provide interesting targets for pre‐clinical testing. Inhibitors to the focal adhesion pathway, as one example, may provide an insight into a new realistic therapeutic approach to improve outcome in *CRLF2*‐r ALL.

## Supporting information

Supporting Information Figures.

Supporting Information Tables.

Supporting Information Table 3.

Supporting Information Table 4.

Supporting Information Table 5.

Supporting Information Table 6.
