# Supplementary material for: Characterisation of the genomic landscape of CRLF2‐rearranged acute lymphoblastic leukemia
Source: Genes Chromosomes Cancer. 2017 Jan 18;56(5):363–72. doi: 10.1002/gcc.22439 (PMC5396319; doi:10.1002/gcc.22439)
Supplement: Supplementary file 1 — Supporting Information Figures. [file GCC-56-363-s001.doc]

**
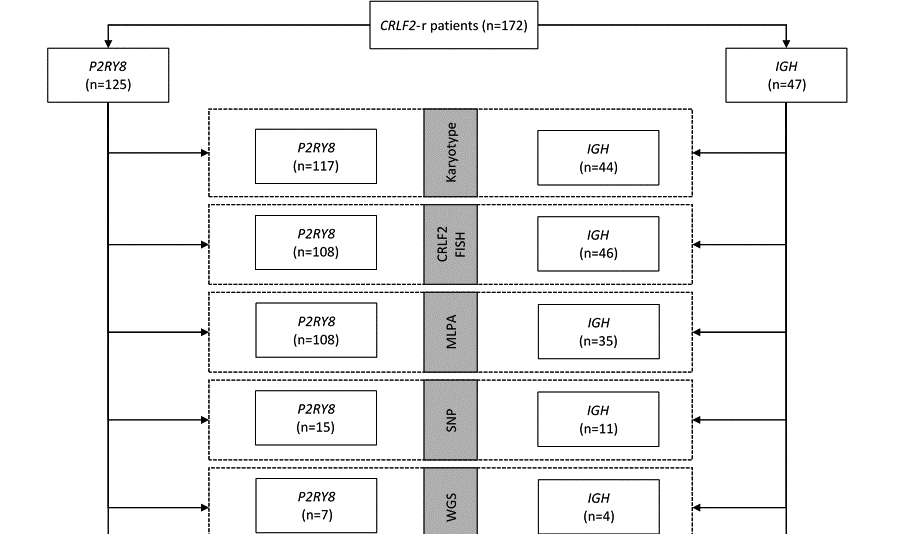
**

**Supplementary Figure 1. Consort diagram**

Analysis profile for investigation of the genomic landscape of patients with *CRLF2*-d ALL. A total of 172 patients were indentified to have either a *IGH-CRLF2* (n=47) or a *P2RY8-CRLF2* (n=125) rearrangement. Karyotypes were available for 44 and 117 patients, respectively. Fixed material was available from 46 and 108 patients, respectively. DNA was available from 35 *IGH* and 108 *P2RY8* patients for MLPA, with 11 and 15 of these patients having enough DNA for SNP arrays, respectively. WGS and WES was performed on 4 patients with IGH and 7 with *P2RY8* involvement with a further 16 and 53 patients undergoing targetted sequencing for *JAK* mutations.


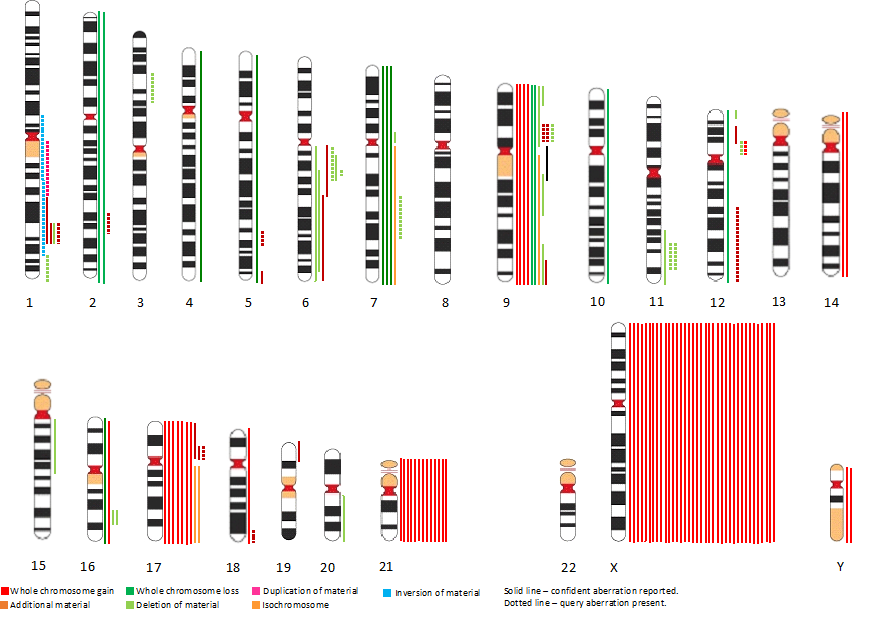


**Supplementary Figure 2. Ideogram of cytogenetic rearrangements reported in the karyotypes of patients with *CRLF2-*r ALL.**

Ideogram of chromosomal abnormalities observed in 83 patients with *CRLF2*-r (26 patients with had reciprocal translocations only and are therefore not depicted in this figure). Somatic numerical gains of chromosome 9 (n=4), 14 (n=3), 17 (n=7), 21 (n=13) and X (n=37) were recurrent in patients with *CRLF2-r* ALL [excluding gains involved in high hyperdiploidy and near tetraploid karyotypes]. Aneuploidy of chromosomes 9 was present in patients with *P2RY8-CRLF2* only, although this did not reach statistical significance (7% v 0%, p=0.089). When investigating different combinations of chromosome aneuploidy, no two specific chromosomes were associated with either *IGH-CRLF2* or *P2RY8-CRLF2*. Interestingly of the seven patients with dic(9;20), all had involvement of *P2RY8-CRLF2*, however this did not reach statistical significance due to low patient numbers. Deletions involving 6q and 9p and additional material located at 1q were rare, but recurrently observed.


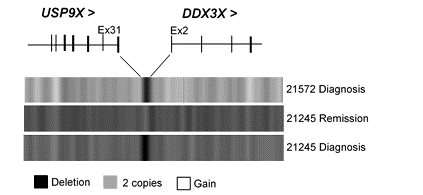


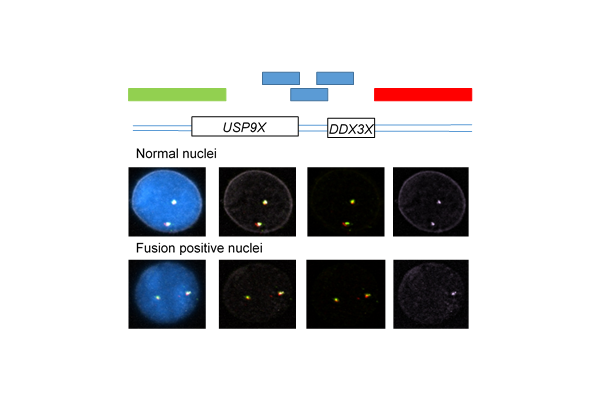


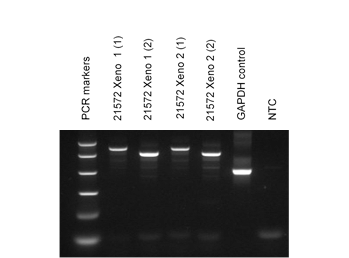


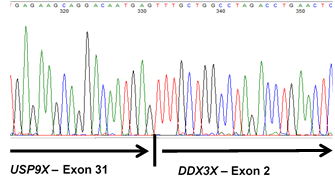


**Supplementary Figure 3. Characterisation of the novel fusion, *USP9X-DDX3X*.** (a) Heat map generated from SNP 6.0 data using the Affymetrix Genotyping Software to show the presence of an interstitial deletion leading to the fusion of sequences from *USP9X* to *DDX3X* in 2 patients (one with a matched remission sample). The deletion is present only in the diagnostic samples. (b) Diagram showing the location and fluorophores for a three colour FISH probe used to screen additional *CRLF2*-d patients for the presence of this fusion. Top panel shows a normal interphase nucleus hybridised with the FISH probe. From left to right: Two complete three colour fusions and DAPI staining; two complete three colour fusions; two red and green fluorophores only; two aqua fluorophores only. Bottom Panel shows a fusion positive nucleus with the far right image showing loss of one aqua probe which lies over the region of deletion, confirming the presence of the fusion in this cell. Due to the subclone nature of this rearrangement, this fusion would not be visible by SNP array analysis in all patients (c) Agarose gel of RT-PCR detection of the fusion products using 2 sets of primers; (1) *USP9X* exon 31 and *DDX3X* exon 6; (2) *USP9X* exon 31 and *DDX3X* exon 5. RNA obtained from patient derived xenograft cells (2 mice, xeno 1 and xeno 2) originating from a fusion positive patient.. The fusion is expressed in both samples. The bands were cut out, purified and sent for sequencing. (d) Sequencing results showing the exact breakpoint within both genes. This fusion results in the juxtaposition of exon 31 of *USP9X* to exon 2 of *DDX3X*.


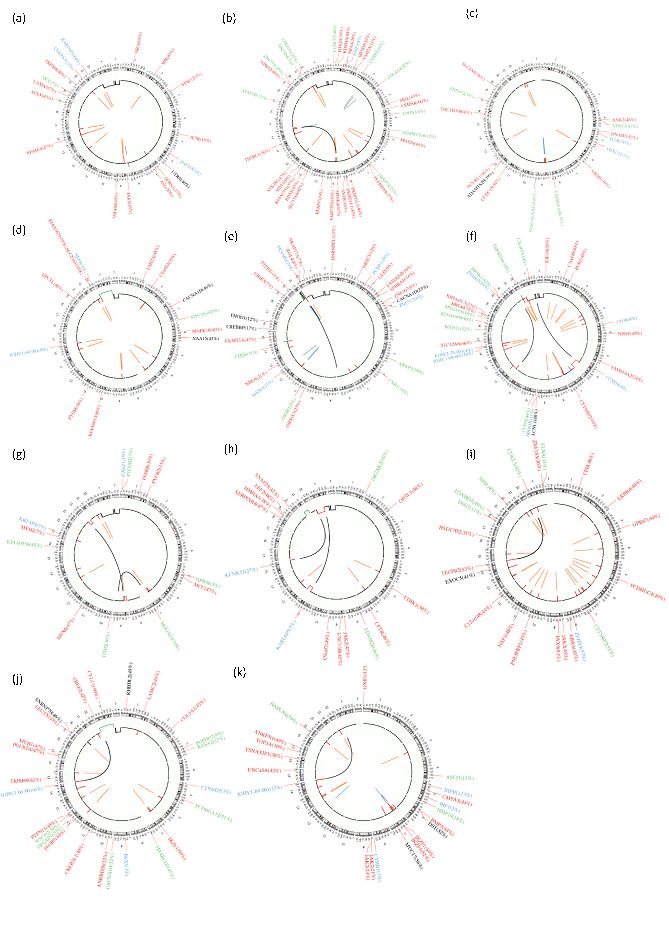


**Supplementary Figure 4. Circos plot for seven *P2RY8-CRLF2* and four *IGH-CRLF2* patients.**

(a-g) Patient with *P2RY8-CRLF2* - 9534, 11538, 20638, 20753, 11706, 21991 and 21819 (h-k) and patients with *IGH-CRLF2* - 11543, 21245, 21470 and 19599. The first (outer) ring displays the names of gene where point mutations and in/dels were identified by WES. The colour of the gene name infers the type of mutation detected; red - missense mutation; green - silent mutation; blue - insertion/deletion; black - nonsense mutation. The variant allele frequency is noted in parenthesis. The second ring shows chromosomal positions. The third ring show CNA detected by SNP6.0 arrays. Black shows a normal copy number of 2, red a copy number loss (copy number 1, shorter line or 0, longer line) and green a copy number gain (copy number of 3, shorter line or 4, longer line). The thickness of the line reflects the size of the effected area. The fourth (inner) ring shows the validated SV detected by paired-end sequencing. The coloured lines depict the following rearrangement types; black – translocation, orange – deletion, blue – inversion, purple – tandem duplication.
