# Supplementary material for: Characterisation of the genomic landscape of CRLF2‐rearranged acute lymphoblastic leukemia
Source: Genes Chromosomes Cancer. 2017 Jan 18;56(5):363–72. doi: 10.1002/gcc.22439 (PMC5396319; doi:10.1002/gcc.22439)
Supplement: Supplementary file 3 — Supporting Information Table 3. [file GCC-56-363-s003.docx]

**Supplementary Table 3:** FISH probe combinations, clone details and cut off values used

| **Target Genes** | **Probe combination** | **Cut off %** |
| --- | --- | --- |
| *ADD3* | CTD – 2511A8 (SG) + | 7 |
|  | 10 centromere (SR) Cytocell |  |
| *PBX3* | W12-922N6 (SG) + W12-1320F14 (SG) | 8 |
|  | + 9 centromere (SR) Cytocell |  |
| *SERP2-TSC22D1* | W12-166J21 (SG) + W12-1982K22 (SG) + | 7 |
|  | W12-2958F19 (SG) + RBI (SR) Abbott Molecular |  |
| *USP9X-DDX3X* | RP11-842M22 (SG) + RP11-10K13 (SR) + | 9* |
|  | W12-2854F4 (SAq) + W12-894E16 (SAq) + |  |
|  | W12-2762G13 (SAq) |  |
| *SLX4IP* | W12-516O3 (SG) + RP11-290F20 (SR) | 9 |

**Abbreviations:** SGd, spectrum gold; SR, spectrum red; SG, spectrum green

* When scoring the cut off slides we never observed the signal pattern indicative of the fusion. The abnormal signal patterns observed were always loss or gain of fusions or signals that were broken apart.
