# Supplementary material for: Characterisation of the genomic landscape of CRLF2‐rearranged acute lymphoblastic leukemia
Source: Genes Chromosomes Cancer. 2017 Jan 18;56(5):363–72. doi: 10.1002/gcc.22439 (PMC5396319; doi:10.1002/gcc.22439)
Supplement: Supplementary file 4 — Supporting Information Table 4. [file GCC-56-363-s004.docx]

**Supplementary** **Table 4.** Clinical data for all patients with *CRLF2*-r ALL, and a comparison between those with *P2RY8* or *IGH* involvement.

|  | **Total** | ***P2RY8*** | ***IGH*** | **p-value** |
| --- | --- | --- | --- | --- |
| **Total** | 172 | 125(73) | 47(27) |  |
| **Sex** |  |  |  | 0.862 |
| Female | 70(41) | 50(40) | 20(43) |  |
| Male | 102(59) | 75(60) | 27(57) |  |
| **WBC*** |  |  |  | **0.016** |
| <50 | 120(70) | 94(75) | 26(57) |  |
| >50 | 51(30) | 31(25) | 20(43) |  |
| Median | 22 | 20 | 35 |  |
| **Age** |  |  |  | **<0.001** |
| <10 | 123(71) | 106(85) | 17(36) |  |
| Oct-15 | 20(11) | 8(6) | 12(26) |  |
| 18-24 | 15(8) | 5(4) | 10(21) |  |
| 25+ | 14(8) | 6(5) | 8(17) |  |
| Median | 5 | 4 | 14 |  |
| **NCI Risk** |  |  |  | **<0.001** |
| SR | 88(51) | 77(62) | 11(23) |  |
| HR | 84(49) | 48(38) | 36(77) |  |
| **DS-ALL** |  |  |  | **0.087** |
| No | 111(69) | 76(65) | 35(80) |  |
| Yes | 50(31) | 41(35) | 9(20) |  |
| **MRD #** |  |  |  | 1 |
| -ve | 18(33) | 13(33) | 5(33) |  |
| +ve | 36(67) | 26(67) | 10(67) |  |

**Abbreviations:** WBC, WBC; * 1 patients WBC is missing; SR, standard risk; HR, high risk; DS-ALL, Down syndrome ALL; # only UKALL2003 patients.
