# Supplementary material for: Characterisation of the genomic landscape of CRLF2‐rearranged acute lymphoblastic leukemia
Source: Genes Chromosomes Cancer. 2017 Jan 18;56(5):363–72. doi: 10.1002/gcc.22439 (PMC5396319; doi:10.1002/gcc.22439)
Supplement: Supplementary file 5 — Supporting Information Table 5. [file GCC-56-363-s005.docx]

**Supplementary Table 5 –** Comparison of clinical and genetic features between patients with *CRLF2*-r ALL, with and without Down syndrome.

|  | **Total*** | **Other** | **DS-ALL** | **p-value** |
| --- | --- | --- | --- | --- |
| **Total** | **161** | **111(69)** | **50(31)** |  |
| Sex |  |  |  | 0.299 |
| Female | 66(41) | 49(44) | 17(34) |  |
| Male | 95(59) | 62(56) | 33(66) |  |
| iAMP21 |  |  |  | **0.003** |
| No | 145(90) | 95(86) | 50(100) |  |
| Yes | 16(10) | 16(14) | 0(0) |  |
| +X |  |  |  | 0.146 |
| No | 106(66) | 78(70) | 28(57) |  |
| Yes | 54(34) | 33(30) | 21(43) |  |
| 21 |  |  |  | 0.188 |
| No | 140(89) | 94(86) | 46(94) |  |
| Yes | 18(11) | 15(14) | 3(6) |  |
| 9 |  |  |  | 0.588 |
| No | 154(97) | 107(98) | 47(96) |  |
| Yes | 4(3) | 2(2) | 2(4) |  |
| 14 |  |  |  | 1 |
| No | 154(97) | 106(97) | 48(98) |  |
| Yes | 4(3) | 3(3) | 1(2) |  |
| 17 |  |  |  | 0.704 |
| No | 150(95) | 104(95) | 46(94) |  |
| Yes | 8(5) | 5(5) | 3(6) |  |
| *IL7RA* |  |  |  | 0.133 |
| No | 8(80) | 6(100) | 2(50) |  |
| Yes | 2(20) | 0(0) | 2(50) |  |
| *JAK2*_Ex14 |  |  |  | 0.613 |
| No | 45(62) | 31(65) | 14(56) |  |
| Yes | 28(38) | 17(35) | 11(44) |  |
| *JAK1*_Ex14 |  |  |  | 1 |
| No | 31(86) | 16(89) | 15(83) |  |
| Yes | 5(14) | 2(11) | 3(17) |  |
| *BTG1* |  |  |  | 0.118 |
| No | 118(86) | 77(82) | 41(93) |  |
| Yes | 20(14) | 17(18) | 3(7) |  |
| *CDKN2A/B* |  |  |  | 0.193 |
| No | 86(62) | 55(59) | 31(70) |  |
| Yes | 52(38) | 39(41) | 13(30) |  |
| *EBF1* |  |  |  | 0.502 |
| No | 128(93) | 86(91) | 42(95) |  |
| Yes | 10(7) | 8(9) | 2(5) |  |
| *ETV6* |  |  |  | 0.425 |
| No | 120(87) | 80(85) | 40(91) |  |
| Yes | 18(13) | 14(15) | 4(9) |  |
| *RB1* |  |  |  | 0.225 |
| No | 125(91) | 83(88) | 42(95) |  |
| Yes | 13(9) | 11(12) | 2(5) |  |
| *IKZF1* |  |  |  | **0.001** |
| No | 78(57) | 44(47) | 34(77) |  |
| Yes | 60(43) | 50(53) | 10(23) |  |
| *PAX5* |  |  |  | **0.047** |
| No | 97(70) | 61(65) | 36(82) |  |
| Yes | 41(30) | 33(35) | 8(18) |  |

* 11 cases DS-ALL information is unknown

Red text denotes statistical significance where p<0.05
