# Supplementary material for: Characterisation of the genomic landscape of CRLF2‐rearranged acute lymphoblastic leukemia
Source: Genes Chromosomes Cancer. 2017 Jan 18;56(5):363–72. doi: 10.1002/gcc.22439 (PMC5396319; doi:10.1002/gcc.22439)
Supplement: Supplementary file 6 — Supporting Information Table 6. [file GCC-56-363-s006.docx]

**Supplementary Table 6.** Recurrent CNA identified by MLPA and SNP arrays

|  | **Total** | ***P2RY8*** | ***IGH*** | **p-value** |
| --- | --- | --- | --- | --- |
| **Total** | 172 | 125(73) | 47(27) |  |
| ***CDKN2A/B*** |  |  |  | 0.232 |
| Normal | 89(62) | 64(59) | 25(71) |  |
| Deleted | 54(38) | 44(41) | 10(29) |  |
| ***BTG1*** |  |  |  | **0.004** |
| Normal | 122(85) | 98(91) | 24(69) |  |
| Deleted | 21(15) | 10(9) | 11(31) |  |
| ***EBF1*** |  |  |  | 0.259 |
| Normal | 133(93) | 102(94) | 31(89) |  |
| Deleted | 10(7) | 6(6) | 4(11) |  |
| ***ETV6*** |  |  |  | 0.249 |
| Normal | 124(87) | 96(89) | 28(80) |  |
| Deleted | 19(13) | 12(11) | 7(20) |  |
| ***IKZF1*** |  |  |  | **<0.001** |
| Normal | 82(57) | 72(67) | 10(29) |  |
| Deleted | 61(43) | 36(33) | 25(71) |  |
| ***PAX5*** |  |  |  | 0.295 |
| Normal | 99(69) | 72(67) | 27(77) |  |
| Altered* | 44(31) | 36(33) | 8(23) |  |
| ***RB1*** |  |  |  | 0.746 |
| Normal | 129(90) | 98(91) | 31(89) |  |
| Deleted | 14(10) | 10(9) | 4(11) |  |
| ***USP9X-DDX3X*** |  |  |  | 0.471 |
| Normal | 44(81) | 29(85) | 15(75) |  |
| Deleted | 10(19) | 5(15) | 5(25) |  |
| ***ADD3*** |  |  |  | **0.008** |
| Normal | 43(75) | 33(87) | 10(53) |  |
| Deleted | 14(25) | 5(13) | 9(47) |  |
| ***SERP2 & TSC22D1*** |  |  |  | 0.682 |
| Normal | 48(87) | 32(89) | 16(84) |  |
| Deleted | 7(13) | 4(11) | 3(16) |  |
| ***PBX3*** |  |  |  | 0.752 |
| Normal | 40(71) | 28(74) | 12(67) |  |
| Deleted | 16(29) | 10(26) | 6(33) |  |
| ***SLX4IP*** |  |  |  | 0.309 |
| Normal | 31(70) | 21(78) | 10(59) |  |
| Deleted | 13(30) | 6(22) | 7(41) |  |
|  |  |  |  |  |
| * *PAX5* intragenic amplifications were included with the deletions as they are predicted to be functionally equivalent | | | | |
